# EO771, is it a well‐characterized cell line for mouse mammary cancer model? Limit and uncertainty

**DOI:** 10.1002/cam4.3295

**Published:** 2020-10-07

**Authors:** Augustin Le Naour, Adrien Rossary, Marie‐Paule Vasson

**Affiliations:** ^1^ UMR 1019 Human Nutrition Unit, ECREIN team University of Clermont Auvergne, INRAE, CRNH‐Auvergne Clermont‐Ferrand France; ^2^ Department of Nutrition Gabriel Montpied University Hospital, Jean Perrin Cancer Centre Clermont‐Ferrand France

**Keywords:** antineoplastic agents, breast neoplasms, hormonal receptors, mice, murine cell line, inbred C57BL

## Abstract

Among mouse mammary tumor models, syngeneic cell lines present an advantage for the study of immune response. However, few of these models are well characterized. The tumor line EO771 is derived from spontaneous breast cancer of C57BL/6 mice. These cells are widely used but are referenced under different names: EO771, EO 771, and E0771. The characteristics of the EO771 cells are well described but some data are contradictory. This cell line presents the great interest of developing an immunocompetent neoplastic model using an orthotopic implantation reflecting the mammary tumors encountered in breast cancer patients. This review presents the phenotype characteristics of EO771 and its sensitivity to nutrients and different therapies such as radiotherapy, chemotherapy, hormone therapy, and immunotherapy.

## Graphical abstract

The EO771 cell line presents a molecular pattern ERα‐, ERβ +, PR+ and ErbB2+ and could be considered as a luminal B subtype. The EO771 tumour‐bearing mouse model presents the great interest of developing an immunocompetent mammary neoplastic model using an orthotopic injection.

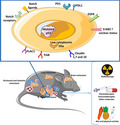

## 1 INTRODUCTION

Breast cancer is the most common cause of cancer deaths among women (522 000 deaths, WHO 2013) and the most frequently diagnosed woman cancer in the world.^1^ Many studies are interested in breast cancer (348,598 results for “mammary cancer” and 405,294 results for “breast cancer” on pubmed, 7 April 2020). This cancer is subdivided into several types, which are well characterized taking into account the cell type of origin, its mutations and its gene expression profile. Before performing clinical trials, experimental models in vitro and in vivo must be used. Thus in preclinical approaches, it is important to choose the right experimental model to know what type of tumor is studied and what type of patient this model could match.

For in vivo experimental approaches, the use of murine models is often done. Among the mice models, the C57BL/6 strain seems to be the most used (about 300 000 publications are found during a pubmed search, 7 April 2020, depending on the way it is spelled) in front of the BALB/c strain (about 200 000 publications found on pubmed, 7 April 2020). The C57BL/6J mouse is the most widely used inbred strain and the first to have genome sequenced.^2^ Although this strain is refractory to many tumors, it is a permissive background for maximal expression of most mutations. However, despite this massive use, few breast tumor lines result from C57BL/6 genetic background. Indeed, only 10 lines are derived from C57BL/6 mice (Table 1): 34T,^3^ AT‐3,^4^ EO771^5^ and its derivative EO771.LMB,^6^ M158,^7^ MG1361,^8^ MMT060562,^9^ Py230,^10^ Py8119,^10^ WT145,^11^ and WT276.^11^ Of the 10 lines, only the EO771 line and its derivated line EO771.LMB come from spontaneous tumors, not induced by the addition of a transgene (as 34T, AT‐3, M158, MG1361, MMT060562, Py230, and Py8119) or a chemical agent DMBA (7,12‐dimethyl‐benzanthracene) (as WT145 and WT276).

**TABLE 1:** C57BL/6 mammary cancer cell lines

Mouse mammary cancer cell lines	Mouse strains	Tumor induction	References
EO771	C57BL/6	Spontaneous	Sugiura and Stock.^5^
EO771.LMB *derived from EO771*	C57BL/6	Spontaneous	Johnstone *et al* ^6^
AT‐3	C57BL/6 MMTV‐PyMT	Transgene addition	Stewart and Abrams.^4^
34T	C57BL/6 × 129/SvJ	Transgene addition	Upadhyay *et al* ^3^
M158	CD‐1 x C57BL/6J MMTV‐c‐myc transgenic mice	Transgene addition	Stewart *et al* ^7^
MG1361	(C57BL/6xDBA)F1 x CD‐1 MMTV‐neu transgenic	Transgene addition	Sacco *et al* ^8^
MMT060562	(C57BL/6xDBA)F1 x CD‐1 MMTV‐neu transgenic	Transgene addition	Akatsu *et al* ^9^
Py230	C57BL/6 MMTV‐PyMT	Transgene addition	Gibby *et al* ^10^
Py8119	C57BL/6 MMTV‐PyMT	Transgene addition	Gibby *et al* ^10^
WT145	C57BL/6J	Chemical agent DMBA	Zinser *et al* ^11^
WT276	C57BL/6J	Chemical agent DMBA	Zinser *et al* ^11^

Note

Breast tumour lines resulted from C57BL/6 genetic background. Among them, only the EO771 line and its derivated line EO771. LMB come from spontaneous tumours. The 34T, AT‐3, M158, MG1361, MMT060562, Py230, and Py8119 cell lines were obtained by addition of a transgene. WT145 and WT276 cell lines were obtained by exposure to the chemical agent DMBA (7,12‐dimethyl‐benzanthracene).

C57BL/6J is the parental substrain; “J” is the laboratory code for The Jackson Laboratory.

Therefore, this review is dedicated to the EO771 mammary cancer cell line. According to the spelling used to search for this lineage in pubmed, numbers of associated publications found are different. A total of 122 publications are identified following pubmed research on 7 April 2020 (Table 2). However, publications using these cells but not indicating the lineage in their title or abstract are not identified.^12^, ^13^, ^14^ These cells have been used for many years and the publication of Sugiura and Stock^5^ from 1952 is frequently presented as the original publication, but earlier articles using this line exist as Homburger's (1948).^15^

**TABLE 2:** Various spelling used to identify EO771 mammary cancer cell line in literatur

Mouse mammary cancer cell lines	Number of Pubmed results
EO 771	25*
EO771	38
E0771	79
EO771.LMB	1

According to the spelling used the numbers of associated publications found are different. A total of 122 publications are identified following pubmed research on 7 April 2020.

* EO 771:25 results but 5 do not concern the tumor line. Research online 7 April 2020.

## 2 EO771 CELL PHENOTYPE

Despite their isolation in the late 1940s, characterization of hormonal receptors and classification of EO771 line remains controversial. Indeed, its classification diverges according to the authors, which is considered as triple negative in 10 publications^6^, ^12^, ^16^, ^17^, ^18^, ^19^, ^20^, ^21^, ^22^, ^23^ and as ERα+ in 19 publications.^13^, ^24^, ^25^, ^26^, ^27^, ^28^, ^29^, ^30^, ^31^, ^32^, ^33^, ^34^, ^35^, ^36^, ^37^, ^38^, ^39^, ^40^, ^41^ Some authors prefer to mention the unclear status of this lineage.^42^, ^43^ Other publications do not report information on the expression of ERα. However, among the 30 publications considering EO771 cells as triple negative or ERα+, only 3 articles analyzed the expression of ERα.^6^, ^40^, ^41^ Contreras‐Zárate *et al* have considered EO771 cells as triple negative and have showed that the proliferation of EO771 cells is independent of the estradiol presence.^23^ But in the same time, they showed that estradiol drives the signaling for brain metastasis of EO771 cells.^23^ Therefore, Hiraga *et al* have observed that the gene encoding ERα is transcribed in EO771 cells^41^ and Gu *et al* have observed the protein expression of ERα by western blot.^40^ However, they observed that this ERα expression is much weaker than that found in MCF‐7 cells (considered as ERα+). In addition, Johnstone *et al* have observed ERα by immunohistochemistry in EO771 cells but have considered these cells as ERα‐ because this receptor is only found in the cytoplasm but not in the nuclear compartment^6^ which is the localization found in primary human breast cancers. Thus, based on these publications, the cells could be considered as ERα‐ because the expression of this receptor is very weak and not at the nuclear level.

Few publications have investigated the status of ERβ, the progesterone receptor and ErbB2 in EO771 cells. The immunohistochemical analysis performed by Johnstone *et al* on primary EO771 and EO771.LMB tumors did not detect the expression of ERβ.^6^ Thus, Hiraga *et al* and Johnstone *et al* have not found gene transcription or protein expression of the progesterone receptor.^6^, ^41^ Similarly, the ErbB2 status for this line is poorly described and contradictory. Indeed, Johnstone *et al* have not found expression of ErbB2 by immunohistochemistry in the tumors formed after injection of EO771 cells^6^ in vivo whereas Hiraga *et al* and Zou *et al* found respectively a transcription of ErbB2 and a protein expression highlighted by westernblot.^44^

As for hormone receptors, the expression of claudin, a marker for the classification of triple negative cancers, is poorly determined. Some authors consider this lineage as claudin‐low without checking it.^31^ On the contrary, Bousquenaud *et al* have found an expression of claudin 1, 7, and 10 in EO771 cells.^28^ However, their expressions are greatly decreased when the mice are fed with a high‐fat diet (HFD), and are associated with a decrease in estrogen receptor and ErbB2 expression. This suggests that a hyperlipid diet would induce a triple negative phenotype for EO771 cells.^28^

**FIGURE 1:**
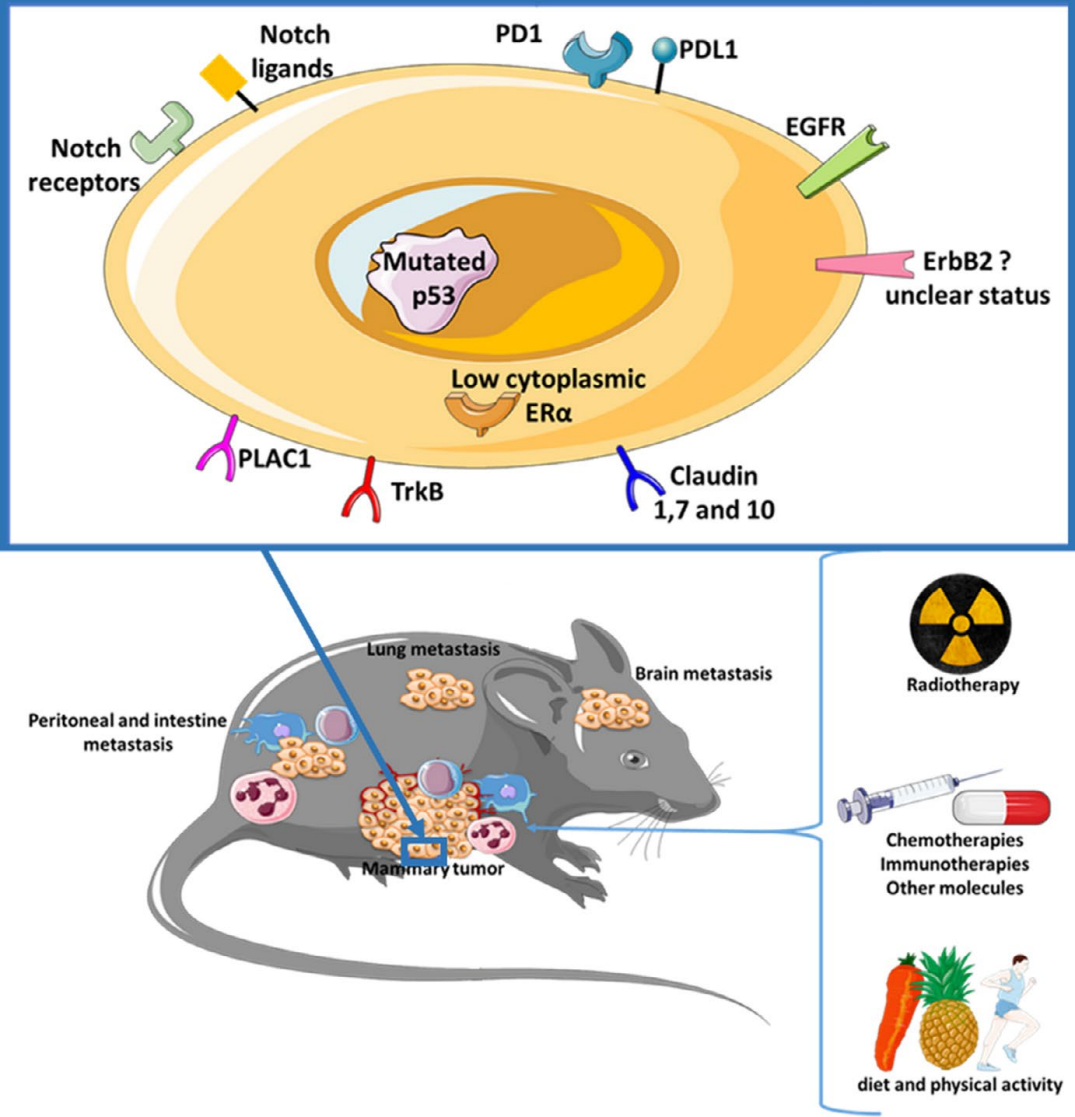
Characteristics of the EO771 cell line and tumor bearing in C57BL/6 mice. Numerous publications have made it possible to refine the phenotype of the EO771 line. This cell line could be considered as a luminal B type mammary cancer. Tumor‐bearing mice models are well‐known and sensitive to explore numerous therapeutic strategies. After injection of EO771 cells, the major metastatic dissemination sites are in the brain, the lung, the intestine, and the peritoneal cavity

Finally, the expression of many other markers from EO771 cells has been characterized such as p53, Notch receptors and ligand, EGFR, PD1 (Programmed Cell Death 1), PDL1 (Programmed Cell Death Ligand 1), *etc* (Table 3, Figure 1).

- Modified EO771 cell lines

**TABLE 3:** Characterisation of hormonal receptors and protein patterns of EO771 line in literature

Biomarker	Expressed	Low to moderate expression	Not expressed	References
Estrogen receptor alpha (ERα)	ER‐α		ER‐α	Hiraga *et al*,^41^ Gu *et al* ^40^ Johnstone *et al* ^6^
Estrogen receptor beta (ERβ)			ER‐β	Johnstone *et al* ^6^
Progesterone receptor (PR)			PR	Johnstone *et al* ^6^
Epidermal Growth Factor Receptor‐2 (ErbB2)	ErbB2		ErbB2	Hiraga *et al*,^41^ Zou *et al* ^44^ Johnstone *et al* ^6^
Claudin	Claudin 1, 7, 10 *decreased by HFD*			Bousquenaud *et al* ^28^
Tumor suppressor	Mutant p53			Johnstone *et al* ^6^
Notch receptors	Notch 2 Notch 3 Notch 4	Notch 1 *but increased by leptin*		Battle *et al* ^24^
Notch ligands	Jagged 1 Delta‐like 4			Battle *et al* ^24^
Matrix Metalloprotease		MMP4		Ager *et al* ^113^
MHC‐I molecules		H‐2Kb H‐2Db		Tu *et al* ^46^
Ligands for NK activation		Clr‐b RAE1		Tu *et al* ^46^
Biomarker for breast cancer	Placental‐specific protein 1 (PLAC1)			Yuan *et al* ^111^
Histamine receptors			Receptor H1 Receptor H2	Vila‐Leahey *et al* ^101^
BDNF/TrkB's oncogenic pathway	TrkB		TrkA TrkC	Contreras‐Zárate *et al* ^23^
Epidermal growth factor receptor	EGFR		EGFR	Johnstone *et al* ^6^ Contreras‐Zárate *et al* ^23^
Cytokeratin 5/6			KRT5/6	Johnstone *et al* ^6^
Immunomodulator protein	PD1 PDL1			Gray *et al* ^20^

List of publications with partial determination of proteins and hormonal receptors expression. Characterisation of phenotype and the expression of many other markers from EO771 cells were generally not done. Among the 30 publications considering EO771 cells, only 3 articles analysed the expression of ERα^6^, ^40^, ^41^. That’s why classification of EO771 line remains controversial.

The EO771 cell line can be genetically modified to follow their growth by fluorescence, for example by expression of green fluorescent protein (GFP),^13^, ^14^, ^23^, ^33^, ^45^ or luciferase^13^ as well as to modify the expression of genes by the CRISPR/Cas9 technology such as the EO771 line devoid of MHC I or MHC II or expressing the tumor antigen NY‐BR‐1.^46^, ^47^

## 3 EO771 MAMMARY TUMOR MOUSE MODELS

The C57BL/6J mouse is the most widely used inbred strain and the first to have genome sequenced.^2^ Although this strain is refractory to many tumors, it is a permissive background for maximal expression of most mutations. It is also susceptible to diet‐induced obesity, type 2 diabetes, and atherosclerosis (https://www.jax.org/strain/000664). Due to the wide use of this mouse strain, many modified strains exist (transgenic, knock out (KO), knock down (KD), overexpression, *etc*) to evaluate tumor development under various conditions. Indeed, the advantage of having a tumor line from the most used mouse strain, C57BL/6, makes it possible to have a lot of genetically modified models of this strain. Thus, EO771 cell tumor growth can be evaluated i) in KO mice for E2 (estradiol),^23^ apolipoprotein E and aromatase,^42^ IFN‐γ receptor,^48^ natural killer lytic‐associated molecule (NKLAM),^33^ MMP13,^49^ calcium‐independent phospholipase A2 β,^50^ IGFBP‐3,^21^, ^51^, ^52^ DUSP1,^53^ GDF2,^54^ BMP10,^54^ and mKIAA1462^55^ but also ii) in transgenic models such as mice overexpressing IL‐15,^56^ obese,^13^, ^35^ NKC^KD^ (exhibiting silenced Ly49 expression in NK cells),^46^ GFP‐LC3 (LC3 linked to GFP expressing mice),^57^ mice transgenically modified to express the HER2 proto‐oncogene (ERBB2)^58^ or FAT‐1 (gene leading to the endogenous formation of ω3‐Polyunsaturated fatty acid from ω6‐Polyunsaturated fatty acid).^44^, ^59^

The use of EO771 cells in mouse models has the advantage of having tumor uptake close to 100%, in particular when an orthotopic mammary implantation is used.^33^ This model is really close to human breast cancer (Figure 1). In reality, mammary cell line EO771 (5 × 10^5^ cells) in suspension into Matrigel™ Matrix orthotopically transplanted into the fourth right mammary fat pad leads to large tumors within weeks.^38^ In fact, implantation into the mammary gland provides a neoplastic model with a complete microenvironment promoting the intercellular dialogue. Syngeneic models have the advantage of being immunocompetent. Thus, communication between different cell types can be explored including the immune response. Therefore, injection of EO771 cells into C57BL/6 mice provides a model of mammary cancer in which the role of host and tumor immunity can be investigated.

- In vivo proliferation and dissemination capacities of EO771 cells

The tumor proliferation of EO771 cells seems to be similar to other transplantable murine and human tumors, especially in relation to the duration of the S phase.^60^ The size of the EO771 tumor is correlated with the cell density but also with the proportion of necrotic tissue.^60^ However, the growth curve of EO771 tumors is independent of the number of cells injected but formulas have been proposed to estimate tumor growth.^61^ The tumor growth can also be influenced by the characteristics of its host. Indeed, the tumor growth is linked to the weight of the mouse injected with EO771 cells with a higher initial growth rate followed by a faster growth deceleration phase in mice with higher body weight compared to mice with lower body weight.^62^

In addition to their proliferation capacity, the ability of EO771 cells to disseminate has also been studied. In mice, a metastatic spread is found for this cell line, with characteristics similar to the human disease.^63^ The sites of metastatic disseminations are multiple from EO771 tumors. Its preferential release seems to be the lungs^6^, ^12^, ^22^, ^26^, ^28^, ^33^, ^36^, ^49^, ^50^, ^54^, ^59^, ^64^, ^65^, ^66^, ^67^, ^68^ but other localizations such as the peritoneum,^26^, ^36^, ^64^, ^68^ bones,^6^, ^41^ brain,^23^ diaphragm,^36^ and intestines^36^ are observed (Figure 1). In addition, EO771 cells express the CD73 antigen (ecto‐5'‐nucleotidase), associated with a pro‐metastatic phenotype in breast cancer.^63^

- In vivo cellular interactions in EO771 mammary tumor mouse model

Innate immune cells, such as macrophages, neutrophils, and dendritic cells, appear to be relatively uninvolved in the growth of the primary tumor but, on the other hand, appear to be important in the metastatic dissemination. Indeed, tumor‐associated macrophages and neutrophils and tumor‐infiltrating dendritic cells are less present in the metastatic microenvironment after splenectomy and this decrease was associated with a decrease in the number of mammary cancer lung metastases.^67^

Similarly, the role of Natural Killer (NK) cells seems important in the control of mammary tumor development and metastatic dissemination. Indeed, the use of an anti‐NK1.1, causing NK cell depletion, leads to an increase in tumor growth compared to untreated mice.^46^ Similarly, the KO of the NK lytic‐associated molecule (NKLAM), which plays an important role in the cytotoxic activity of NK, causes a very large increase of tumor cells in the blood and lungs of mice after orthotopic injection of EO771 cells, compared to wild‐type (WT) mice for NKLAM.^33^ The effect of NK does not appear to be mediated by Toll‐like receptor 3 because its deficiency has a small impact on the tumor growth of EO771 cells.^69^ Interestingly, a study showing that a prior immunization with the injection of another type of tumor cell (Colon adenocarcinoma line Colon38) resulted in an absence of EO771 tumor growth. This immunoprevention is mediated mainly by NK1.1 positive cells.^70^

Finally, the evolution of T lymphocytes and their role during tumor development is also studied in the model of EO771 tumor‐bearing mice. Thus, an increase in the CD4 + and CD8 + populations is observed when the size of the tumor increases. However, the CD4/CD8 ratio differs during tumor development with a predominance of CD8 + T lymphocytes (cytotoxic lymphocytes) in the early stages whereas CD4 + lymphocytes become predominant in late stages. The accumulation of CD8 + T lymphocytes, which may involve IGFBP‐3 (Insulin‐like Growth Factor Binding Protein‐3), is an immune population with a strong antitumor role as shown by the decrease in tumor growth.^51^ Experiments inducing a CD8 + T cell depletion exacerbates EO771 tumor growth, emphasizing the importance of CD8 + T cells in controlling tumor growth.^71^ Within CD4 + cells, the proportions of population subtypes evolve during tumor development with, in the early stages, a predominant Th1 lymphocyte population, known to stimulate antitumor immune responses, and then evolve into subtypes of Treg, associated with a tolerogenic profile, and Th17 cells, in more advanced stages.^72^

In addition to the applications already described, these cells have also been used with other cell types to study possible interactions. Thus, the interactions of EO771 cells with adipocytes or fibroblasts led to protumoral effects. An increase in EO771 cell proliferation is induced by secretions of senescent fibroblasts^73^ or adipocytes.^74^ Finally, this model has also been used to evaluate the effect of the tumor on memory loss,^75^ muscle damage,^76^ or tumor development when associated with a viral infection.^77^

- Impact of obesity and physical activity in EO771 tumor‐bearing mice

A sedentary lifestyle and obesity are studied because of their association with breast cancer^78^ incidence and recurrence. On the contrary, physical activity is inversely associated with breast cancer risk.^78^ To study these parameters, the use of the immunocompetent model is essential because obesity, inducing chronic low‐grade inflammation, and physical activity are able to modulate the immune system. Therefore, the use of a syngeneic model such as orthotopic injection of EO771 cells in C57BL/6 mice represents a relevant model of breast cancer to study the effect of overweight/obesity and physical activity.

The obesity of the C57BL/6 mice, induced either by taking a HFD^28^, ^32^, ^35^, ^36^, ^51^, ^52^, ^79^ or by genetic alteration,^13^, ^35^ leads to a protumoral effect by increasing tumor growth,^13^, ^28^, ^32^, ^52^ promoting tumor angiogenesis,^28^, ^32^ having an immunomodulatory effect^28^ as well as activating pro‐tumor pathways such as AKT/mTOR.^13^

Nachat‐Kappes *et al* have used an environmental enrichment that leads to spontaneous physical activity in mice.^38^ Environmental enrichment resulted in a decrease in COX‐2, which may suggest a decrease in inflammation, and a decrease in tumor volume and weight associated with a decrease in the proliferation index Ki67.^38^ Physical exercise, whether spontaneous^25^, ^38^ or forced,^31^ leads to a slowing down of tumor growth but also makes it possible to modulate angiogenesis^25^, ^31^ and to increase sensitivity to chemotherapy^25^ compared with controlled mice.

Physical activity also induces changes in the cytokine environment, including adipokines, by increasing the plasma ratio of adiponectin/leptin levels.^38^ This ratio of adipokines is decreased in a situation of obesity, which is a major risk of breast cancer.^78^

- Impact of phytonutrients in EO771 tumor‐bearing mice

Various phytonutrients from usual vegetables have been tested in mammary cancer models using EO771 cells. Bioactive compounds such as naringenin (citrus flavonoid),^80^ [10] ‐Gingerol (a major phenolic constituent of ginger root),^16^ secoisolariciresinol diglucoside (polyphenolic plant lignan),^17^ meroxest (synthetic merosesquiterpenes),^29^, ^30^ EGCG (Epigallocatechin Gallate, a major green tea catechin)^81^ and emodin (a Chinese herb‐derived compound)^34^, ^65^ induced antitumor effects against E0771 cells by several ways such as: 1) inducing cell death^16^, ^80^; 2) inhibiting the cell cycle^16^, ^80^; 3) modulating the immune system including macrophages^17^, ^34^, ^65^ or leukocytes^29^, ^34^; 4) decreasing pro‐angiogenic markers such as expression of VEGF^17^, ^29^, ^81^; 5) modulating signaling pathways such as NF‐κB,^17^, ^81^ IRF4,^34^ STAT6,^34^ and C/EBPβ.^34^

## 4 SENSITIVITY TO DIFFERENT THERAPIES

The sensitivity of the EO771 line has been tested against many treatments such as radiotherapy, cytotoxic agents, antiangiogenics, hormone therapy, immunotherapy, gene therapy, and bioactive compounds.

- Sensitivity to radiotherapy

Breast cancer treatment is multimodal and includes radiotherapy.^82^ Therefore, EO771 cells are exposed to radiotherapy in animal models.^83^, ^84^ The EO771 tumor line seems resistant to irradiation by 30 Gy of Cobalt 60 because, despite a transient effect in tumor cell number, the day after irradiation, the effect is limited to a growth delay without tumor regression.^84^

The radioactivity can also be used as a tracer in EO771 tumor‐bearing C57BL/6 mice, such as the use of ^125^I‐labeled 5‐iodo‐2'‐deoxyuridine, which allows tumor progression to be monitored.^85^, ^86^

- Sensitivity to cytotoxic agents

### 4.1 Inhibitors of topoisomerase II and alkylating agents

Anthracyclines and topoisomerase II inhibitors, are among the conventional chemotherapies used in breast cancer, whether in the United States where AC regimen (A = doxorubicin, (anthracycline) and C = cyclophosphamide) is commonly used, or in Europe where FEC100 (combining F = 5‐fluorouracil, E = epirubicin (anthracycline) and C = cyclophosphamide) is frequently used.^87^ Doxorubicin is tested on EO771 cells *in vitro* ^73^ and in vivo, in mouse models to test its antitumor efficacy and its toxicity. Studies on animal models have shown that this line is sensitive to doxorubicin and that the activity of the latter could be improved either by conjugating it to nanoparticles^27^, ^88^ or peptides^37^, ^89^ or by associating it with other molecules such as IL‐2,^64^ TNF,^90^ rapamycin,^57^ or FTY720 (a sphingosine‐1‐phosphate receptor functional antagonist).^35^ These models also tested the toxicity of doxorubicin in mouse mammary cancer models. This anthracyclin causes skeletal muscle dysfunction and increases mitochondrial H_2_O_2_ production inducing a decrease on the mitochondrial respiratory complex supported by complex I (pyruvate/glutamate/malate) and complex II (succinate) substrates.^76^ This toxicity of doxorubicin, in particular at cardiac level, is not modified with the addition of IL‐2^64^ but is decreased when doxorubicin is conjugated with nanoparticles^27^, ^88^ and peptides^64^, ^89^ or associated with rapamycin.^57^

Cyclophosphamide, an alkylating agent, frequently used in breast cancer therapy as in the FEC100 and AC regimen protocols, leads to an initial significant cytotoxic activity but limited in time as the tumor growth restarts.^60^, ^91^

### 4.2 Other cytotoxic agents

In addition to these commonly used chemotherapies, other cytotoxic drugs can be used in the therapeutic arsenal of breast cancer. Thus, methotrexate, an antifolate, is tested in the mouse model of EO771 mammary cancer showing a sensitivity of this tumor to this drug.^92^ This animal model is also tested for the development of a novel antifolate family as analogues of aminopterin^91^, ^93^, ^94^, ^95^, ^96^, ^97^, ^98^, ^99^ whose antitumor activity is superior to methotrexate in EO771 tumor‐bearing mice.

Antipyrimidic drugs such as cytosine arabinoside^100^ and gemcitabine^101^ are also active on EO771 tumors. The vinca alkaloids, such as vinblastine, navelbine, and vindesine induced increase in animal survival.^98^ Similarly, paclitaxel,^102^ cisplatin,^91^ and melphalan^91^ have antitumor activity against EO771 tumors. In contrast, 5‐fluorouracil appears to be of low activity on EO771 tumors.^91^

Other compounds such as ranitidine,^45^, ^101^, ^103^ a substituted 3‐(5‐imidazo[2,1‐b] thiazolylmethylene)‐2‐indolinones^104^ or EB‐3D (a choline kinase 1 inhibitor)^105^ have shown antitumor effects in EO771 mammary cancer models.

- Sensitivity to antiangiogenic agents

Angiogenesis plays an important role in tumor progression and metastatic dissemination.^106^ Thus, the development of antiangiogenic therapy has generated a great enthusiasm in recent years. The EO771 tumor‐bearing mouse model is used to evaluate the role of angiogenesis and to test molecules modulating angiogenesis, especially since this cell line expresses vascular endothelial growth factor (VEGF) receptors 1 and 2.^40^ Tumor development seems to be dependent on angiogenesis. A decrease in neovascularization is found in a mouse model using mKIAA1462^‐/‐^ mice resulting in a loss of “junctional protein associated with coronary artery disease,” and is associated with a decrease in tumor volume compared with the control group of EO771 tumor‐bearing mice.^55^ Antiangiogenic agents targeting VEGF or its receptors have been tested in this model. The SU11248^39^ is a selective protein kinase inhibitor inducing inhibition of, among others, VEGFR types 1‐3 found in human breast cancer. SU11248,^39^ but also pyrrolidine dithiocarbamate,^40^ in EO771 tumor model modulated neoangiogenesis by decreasing intratumoral microvessel density. This effect is associated with a decrease in tumor weight compared to the control group.^39^, ^40^ The effect of pyrrolidine dithiocarbamate passed in particular through the inhibition of autocrine and paracrine VEGF effects by decreasing its expression and reducing NFkB activation.^40^ This molecule pyrrolidine also inhibited the growth and migration of EO771 cells and had a synergistic effect with the VEGF receptor inhibitor SU5416.^40^ Lu *et al* have showed that EO771 tumor growth is increased by stimulating VEGF‐dependent angiogenesis but that is inhibited by the use of SU5416.^107^ The antioxidant N‐acetylcysteine has also been studied in models using EO771 cells. However, despite the fact that N‐acetylcysteine prevented Hif‐1α stabilization under hypoxia in vitro, it did not reduce in vivo the tumor growth or the survival of EO771 tumor‐bearing mice but rather, increased the metastatic burden.^108^ Finally, vascular endothelial protein tyrosine phosphatase has also been investigated. Inhibition of the latter resulted in a delay in tumor growth during tumor establishment, but had no impact once the tumor is well established.^68^

- Sensitivity to hormone therapy

Since the status of EO771 cells in hormone receptor expression remains controversial, few studies have evaluated the impact of hormone therapy in this model. Johnstone *et al* have observed a reduction in the growth of EO771 tumors during treatment with tamoxifen,^6^ which may therefore suggest an expression of estrogen receptors in these cells.

- Sensitivity to immunotherapy

As previously mentioned, the EO771 tumor‐bearing mouse model is immunocompetent and of interest for evaluating the efficacy of immunotherapies such as the use of cytokines or immune checkpoint inhibitors. Indeed, it represents a model of choice because the EO771 cells express immunomodulatory molecules as PD1 and PDL1 in the basal state.^20^ Moreover, in the presence of IFNγ, the expression of PD1 and PDL1 is increased in EO771 cells^20^ and the overexpression of mucin 1 in these tumor cells resulted in an increase in PDL1 expression.^19^

Therefore, therapies such as immune checkpoint inhibitors have been tested in this model. The use of anticytotoxic T lymphocyte‐associated protein 4 (CTLA4) or antiprogrammed cell death 1 (PD1) therapy allowed an increase in the number of circulating CD8 + T cells and IFN‐γ leading to T lymphocyte‐mediated antitumor response.^48^ However, anti‐PD1 alone leads to partial antitumor activity in the model of EO771 tumor‐bearing mice.^22^ Thus, it seems interesting to associate them with other treatments, such as surgery, chemotherapy, or even other immunotherapies. Liu *et al* have showed that neoadjuvant immunotherapy combining anti‐PD1 and anti‐CD137 enhanced therapeutic efficacy compared to surgery alone or surgery followed by this immunotherapy.^12^ This increase in therapeutic efficacy is associated with an increase in CD8 + lymphocytes in the blood, spleen, liver, and lungs.^12^ However, this neoadjuvant immunotherapy must be performed shortly before surgery because, after 10 days, the benefits of this treatment are lost.^18^ Anti‐PD1 has also been associated with a Tyro3, Axl, and Mertk inhibitor (BMS‐777607), which are tyrosine kinase receptors having immunomodulatory properties. Their association allowed to significantly reduced tumor growth and incidence of lung metastasis, associated with an increase in proinflammatory cytokines and an infiltration of antitumor effector T cells.^22^ The use of phosphatidylserine‐targeting antibodies decreases tumor growth and increases the antitumor efficacy of anti‐PD1 in a syngeneic model using EO771 cells by promoting tumor infiltration of T cells and increasing the production of proinflammatory cytokines.^20^

Proinflammatory cytokines, such as interleukin (IL) 2 and tumor necrosis factor (TNF), have also been investigated in models using EO771. Treatment with IL‐2 resulted in prolonged survival of EO771 tumor‐bearing mice^109^, ^110^ and improved the efficacy of doxorubicin chemotherapy.^64^ This effect required the action of lymphocytes, especially CD8 +.^64^, ^109^, ^110^ Similarly, the presence of TNF led to the stimulation of CD8 + cytotoxic T lymphocytes and NK cells, allowing the complete remission of EO771 tumor‐bearing mice, when this cytokine was associated with doxorubicin chemotherapy.^90^

Other immunomodulatory agents acting on other immune cells have also been studied in the mouse model of EO771 mammary cancer. For example, the use of a Cxcr2 antagonist decreased tumor growth by acting on different immune populations. In fact, it led in the tumor to a decrease in immunosuppressive cells such as myeloid‐derived suppressor cells (MDSC) and regulatory T cells (Treg) and, on the contrary, an increase in antitumor cells such as cytotoxic lymphocytes, NK cells, dendritic cells, and macrophages.^111^ These latter have an ambivalent role on tumorigenesis depending of their polarization. Indeed, M1 macrophages have tumoricidal activity, while M2 macrophages exhibit low amounts of antigen presentation and suppress antitumor immunity.^112^ Treatment with an anti‐MMP14 inhibitory antibody (DX‐2400) increased the tumor‐associated macrophage number and polarized them toward an antitumoral M1 phenotype.^113^

- Sensitivity to gene therapy

Gene therapy has also been tested in the EO771 tumor‐bearing mouse model. A reduction in tumor growth of the primary tumor but also at the metastatic level is observed when using adenovirus inducing the expression “brain‐derived neurotrophic factor”^36^ or MBP‐1^66^ (Myelin Basic Protein 1).

## 5 CONCLUSION

Therefore, the characteristics of the EO771 cells are well described but some data are contradictory. Thus, their molecular classification remains controversial at the present time between the luminal and triple negative subtype, close to the luminal B phenotype. However, the luminal A phenotype could be excluded considering the articles showing them as ERα‐. More accurate phenotyping of this cell line is needed as well as for other murine cell lines since very few of them are clearly defined in terms of expression of estrogen, progesterone, and ErbB2 receptors. The use of this cell line has been carried out in 2D, 3D and in vivo models. 3D cell culture techniques are now a way of using cancer cell lines. Even if there is currently only one publication on this subject,^107^ the use of this line in 3D models is possible. The establishment of 3D models with EO771 cells will provide valuable data for understanding breast cancer.

The EO771 cell line presents the great interest of developing an immunocompetent neoplastic model using an orthotopic injection reflecting the mammary tumors encountered in breast cancer patients. This EO771 tumor‐bearing mouse model has been well characterized considering its sensitivity to various antineoplastic treatments and even other therapic approaches including nutritional interventions and physical activity (Figure 1).

Despite some uncertainties, all these data lead us to consider the EO771 cell line as a very relevant candidate for providing an experimental mammary tumor model very close to human breast cancer.

## CONFLICT OF INTEREST

The authors declare that they have no conflict of interest.

## AUTHOR CONTRIBUTIONS

Augustin Le Naour is the major contributor in writing the manuscript and analyzing the literature. Adrien Rossary and Marie‐Paule Vasson participated in the scientific discussions for the manuscript writing and during the revision of the manuscript. All the authors approved the final version of the manuscript.

## Data Availability

Not applicable.
